# Unmasking and Addressing COVID-19–Related Maladaptive Grief Reactions Among Suicidal Youth: Pilot Evidence for an Enhanced Psychiatry Emergency Room Safety Preventive Intervention

**DOI:** 10.1016/j.jaacop.2024.10.001

**Published:** 2024-10-16

**Authors:** Naser Ahmadi, Julie B. Kaplow, Alan Steinberg, James T. McCracken, Steven J. Berkowitz, Robert S. Pynoos

**Affiliations:** a David Geffen School of Medicine, University of California Los Angeles, Los Angeles, California; b Olive View UCLA Medical Center, Sylmar, California; c Trauma and Grief Center at The Hackett Center for Mental Health, Houston, Texas; d Tulane University School of Medicine, New Orleans, Louisiana; e University of California San Francisco, San Francisco, California; f University of Colorado Anschutz Medical Campus, Denver, Colorado

**Keywords:** COVID-19, emergency room, maladaptive grief reactions, posttraumatic stress disorder, reminder-focused positive psychiatry suicide prevention

## Abstract

**Objective:**

The coronavirus pandemic has resulted in catastrophic levels of mortality among parents and caregivers worldwide. This study explored the benefit of a grief-enhanced trauma-informed safety preventive intervention in enhancing parental support, ameliorating maladaptive grief and posttraumatic stress reactions, and reducing suicidal risk among youth who have experienced a COVID-19–related death of a parent/relative.

**Method:**

This retrospective cohort study included youth with COVID-19–related maladaptive grief and posttraumatic stress disorder (PTSD) reactions and suicidality who received either reminder-focused positive psychiatry with suicide prevention (RFPP-S) (n = 62) or treatment as usual (TAU) (n = 62) at a large county psychiatric emergency room (PER). Clinical assessments were administered at baseline, day 2, and postdischarge weeks 1 and 4.

**Results:**

The prevalence of youth with PTSD and suicidality presenting at the PER was 61%; 40% had COVID-19–related PTSD, of whom 90% also had maladaptive grief reactions. On day 2, a significant reduction was noted in maladaptive grief reactions and PTSD symptoms, reactivity to trauma and loss reminders, guilt feelings, negative affectivity, and avoidance in youth receiving RFPP-S, but not in TAU youth (*p* = .001). RFPP-S, but not TAU, was associated with a significant reduction in Columbia–Suicide Severity Rating Scale scores, decreased length of stay, and rapid acute crisis stabilization (*p* = .001). A substantial increase in well-being, engagement, connectedness with caregivers, resilience and adaptability, posttraumatic growth, and enhanced coping skills was found in RFPP-S youth (*p* < .05), but not in TAU youth. Postdischarge follow-up was significantly enhanced in RFPP-S youth (100%) compared with TAU youth (32%). RFPP-S, but not TAU, was associated with no readmissions for suicidality postdischarge, sustained reduced maladaptive grief reactions and PTSD symptoms, and reduced reactivity to trauma/loss reminders at 1-month follow-up.

**Conclusion:**

This study suggests the feasibility and potential effectiveness of providing a grief-enhanced trauma-informed safety preventive intervention in a PER. This intervention was associated with reducing severity of maladaptive grief and posttraumatic stress reactions, improving mental well-being and youth–caregiver interactions, and promoting safety and acute crisis stabilization. Parental/PER therapeutic support has promise as an important public health strategy for this population of grieving suicidal youth.

**Diversity & Inclusion Statement:**

We worked to ensure sex and gender balance in the recruitment of human participants. We worked to ensure that the study questionnaires were prepared in an inclusive way. We worked to ensure race, ethnic, and/or other types of diversity in the recruitment of human participants. One or more of the authors of this paper self-identifies as a member of one or more historically underrepresented racial and/or ethnic groups in science. We actively worked to promote sex and gender balance in our author group. We actively worked to promote inclusion of historically underrepresented racial and/or ethnic groups in science in our author group. While citing references scientifically relevant for this work, we also actively worked to promote inclusion of historically underrepresented racial and/or ethnic groups in science in our reference list. The author list of this paper includes contributors from the location and/or community where the research was conducted who participated in the data collection, design, analysis, and/or interpretation of the work.

## Plain language summary

COVID-19–related maladaptive grief and posttraumatic stress reactions are prevalent among adolescents with suicidality who visited the psychiatry emergency room. The authors investigate the role of grief-enhanced trauma-informed safety preventive intervention for youth with COVID-19-related maladaptive grief and posttraumatic stress in a psychiatric emergency room setting. The authors found that such intervention in the psychiatric emergency room setting was associated with a robust reduction in maladaptive grief and posttraumatic stress reactions, reactivity to trauma and loss reminders, and suicidality, and resulted in rapid stabilization of acute crisis and favorable clinical outcomes. The authors conclude that parental and emergency room therapeutic intervention holds promise for COVID-19–related grieving suicidal youth.

The coronavirus (COVID-19) pandemic has led to widespread morbidity and mortality, pervasive trauma and maladaptive grief reactions, disruption of a sense of safety, diminished social interactions and connections, and financial insecurity. Approximately 290,000 US youths have experienced the death of a parent or caregiver due to COVID-19 at the time of this writing.^1^ In addition, COVID-19–related deaths among people with mental disorders have been shown to be higher than in the general population, especially in African Americans and individuals with Hispanic ethnicity.^2^^,^^3^ Suicide is the second leading cause of death in youth ages 10 to 19, especially among those experiencing grief and posttraumatic stress disorder (PTSD).^4^^,^^5^

Enhanced psychological and physiological reactivity to trauma and loss reminders (RTLR), a core symptom of both maladaptive grief and PTSD, is associated with increased aggressive behavior, emotional constriction, social withdrawal, impaired fear extinction, diminished neuroplasticity, and increased neurovascular inflammation. RTLR plays a significant role in developing and maintaining maladaptive grief and PTSD and is associated with suicidality and major adverse health outcomes.^6^, ^7^, ^8^

Recent studies have provided evidence of the efficacy of grief-focused and trauma-informed interventions in reducing the severity of PTSD and maladaptive grief reactions, along with favorable clinical outcomes (eg, enhanced mental well-being and social interaction).^9^ A prior study revealed the feasibility of reminder-focused positive psychiatry with suicide prevention (RFPP-S), which treats both youth and caregivers in improving PTSD symptoms by reducing the severity of RTLR, enhancing skills for coping with trauma and loss reminders, improving parent–child interactions, increasing distress tolerance, and acquiring safety skills. These interventions led to a rapid stabilization of acute crisis, adherence to post–psychiatric emergency room (PER) follow-up visits and treatments, and favorable clinical outcomes among adolescents with PTSD and suicidality.^7^^,^^10^^,^^11^

### Aims of This Study

This study evaluated the effect of a brief grief-enhanced trauma-informed safety preventive intervention to ameliorate maladaptive grief and PTSD reactions, reduce the severity of trauma reminder reactions, diminish the risk of suicidality, enhance parental support, and prevent adverse clinical outcomes among adolescents with COVID-19–related maladaptive grief and PTSD with suicidality who received treatment at the Olive View UCLA Medical Center PER.

## Method

### Study Subjects

This retrospective cohort study, using a nested matched case-control design, included youth with COVID-19–related maladaptive grief reactions and PTSD reactions and suicidality who received either reminder-focused positive psychiatry and suicide prevention (RFPP-S) intervention (n = 62), or treatment as usual (TAU) (n = 62) during their Olive View ULCA PER visit.

PER clinicians conducted evaluations using the Columbia–Suicide Severity Rating Scale (C-SSRS), the Patient Health Questionnaire (PHQ-9), and PTSD and grief measures. All consecutive adolescents with COVID-19–related PTSD reactions and maladaptive grief with suicidality (with a C-SSRS score of ≥3 and 12% suicide behavior) who were admitted to the PER and were free of other major psychiatric disorders and prior suicide attempts were provided with developmentally age-appropriate RFPP-S, a grief-enhanced trauma-informed intervention, on Wednesday or Thursday. Controls matched for age, gender, and enrollment time were randomly selected from the pool of eligible adolescents with COVID-19–related PTSD and maladaptive grief with suicidality who were not provided with RFPP-S in the Olive View UCLA PER to form the incidence density sampling risk set for the index case. All subjects were on Medi-Cal, California’s Medicaid insurance program, and were screened, clinically assessed, and received standard of care, the Safe Alternatives for Teens and Youths–Acute (SAFETY-A) intervention. All youth and caregivers received SAFETY-A for risk assessment and safety planning during one 30-minute intervention. The SAFETY-A intervention was provided by 2 clinicians and used an ABCD approach including the following:

• A—Assessment: a behavioral assessment of risk

• B—Building hope and reasons for living: an intervention based on personal and family strength of youth

• C—Connect: an intervention to strengthen connections with health system and other responsible adults who can provide protection and support

• D—Develop a personal safety plan: an intervention for youth to be used when the risk of self-harm is increased^12^

Youth receiving RFPP-S received 2 text messages or phone calls 1 week postdischarge reminding them to attend postdischarge mental health visits.

Demographic and clinical characteristics and clinical outcomes of subjects were obtained using Los Angeles County Department of Health Services/Department of Mental Health clinical electronic medical records, Department of Health Services administrative records (i2b2 data portal; https://www.i2b2.org/), and chart review. Subjects were followed with a mean follow-up of 1 month from September 2020 to November 2021.

### Control Ascertainment

Age-, gender-, and enrollment time–matched controls were randomly selected from the pool of 180 eligible youths with COVID-19–related maladaptive grief reactions and PTSD symptoms and suicidality and without RFPP-S who were admitted to PER on Monday or Tuesday and received standard-of-care treatment, SAFETY-A, at the Olive View UCLA PER, using a nested case-control design.

### Definition of PTSD

PTSD and persistent complex bereavement disorder diagnoses were based on *DSM-5* criteria, using *ICD* and *DSM-5* codes. The onset of PTSD and persistent complex bereavement disorder was more than 6 months before the PER visit for all studied participants.

### Assessments

As a part of clinical practice, the following assessments were administered on day 1, day 2, week 1, and week 4: C-SSRS; UCLA Brief COVID-19 Screen for Child/Adolescent PTSD; UCLA Child/Adolescent PTSD Reaction Index for *DSM-5*; UCLA Trauma Reminder Inventory; UCLA Loss Reminder Inventory; Persistent Complex Bereavement Disorder (PBCD) Checklist; the PHQ-9; Parent-Child Interaction Questionnaire–Revised (PACHIQ-R); and positive batteries including PERMA Positive Emotion, Engagement, Relationships, Meaning, and Accomplishment (PERMA) scale, Posttraumatic Growth Inventory (PTGI), and gratitude, resilience, and parent–child interaction questionnaires.^13^, ^14^, ^15^, ^16^, ^17^, ^18^, ^19^, ^20^, ^21^

### Intervention (RFPP-S)

**Figure 1:**
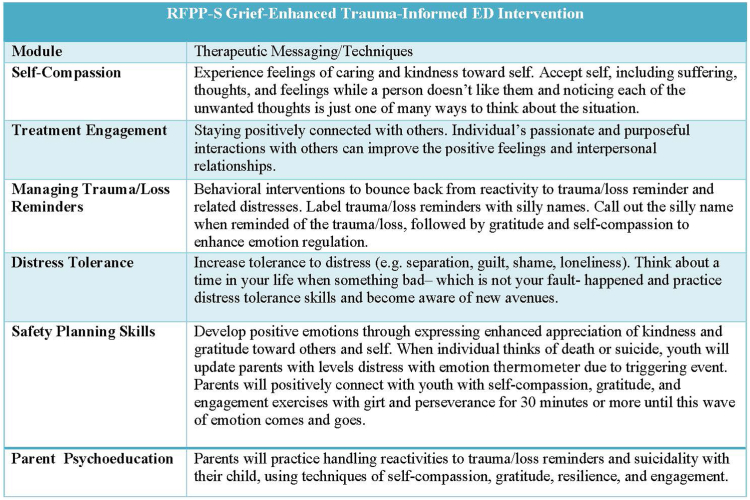
Modules of Reminder-Focused Positive Psychiatry and Reminder-Focused Positive Psychiatry and Suicide Prevention (RFPP-S) Emergency Department (ED) Intervention ***Note:****Self-compassion, treatment engagement, managing trauma/loss reminders, distress tolerance, safety planning skills for youth, and parent psychoeducation are modules of the RFPP-S grief-enhanced trauma-informed ED intervention.*

As previously reported, RFPP-S, a developmentally appropriate reminder-focused trauma/loss-informed and safety prevention intervention, consists of components addressing trauma/loss reminders and avoidance of reminders, negative cognitions, safety planning, and distress tolerance skillsets for adolescents with PTSD and their families (Figure 1). The trauma/loss reminder component enhances recognition of and coping with physiological and psychological reactions to trauma/loss reminders. RFPP-S also increases tolerance of distress (eg, feelings of guilt, shame, loneliness) and promotes moving from reactivity during reminders and related suicidality to experiencing feelings of caring and kindness toward self, the use of behavioral interventions to bounce back from distress with enhanced emotional regulation, staying connected with others, and developing positive emotions through the expression of enhanced appreciation of self and gratitude toward others.

The avoidance and negative cognition components are designed to increase flexible thinking and expressing and evaluating negative thoughts, enhance labeling emotional and physical reactions related to grief and PTSD reactions, increase the use of strategies to promote constructive developmental progression, improve treatment adherence, increase purposeful interactions with parents and interpersonal relationships, and improve safety skills. RFPP-S focuses on enhancing contextual discrimination of trauma and loss reminders (eg, “it’s like what happened, but it’s not happening again”), enhancing emotional regulation, promoting adaptive coping strategies in response to trauma/loss reminders, and enhancing safety skills. All adolescents and their parents participated in RFPP-S.

Using the UCLA Brief COVID-19 Screen for Child/Adolescent PTSD, UCLA PTSD Child/Adolescent Reaction Index for *DSM-5*,^19^ UCLA Trauma Reminder Inventory,^20^ and UCLA Loss Reminder Inventory,^21^ the top 3 reminders associated with the recent suicidal ideation (including COVID-19–related traumatic/losses) were identified. RFPP-S modules were practiced with youth for 10 minutes twice daily for 2 consecutive days. Parents received one 10-minute parent psychoeducation session using RFPP-S modules.

### Outcome End Points

The outcome end points were to assess the change in maladaptive grief reactions and PTSD symptoms and suicidality among adolescents with COVID-19–related grief with PTSD and suicidality after receiving RFPP-S compared with TAU and to compare clinical outcomes in terms of postdischarge mental health follow-up, suicidality, and psychiatric rehospitalization within 30 days postdischarge.

### Statistical Analysis

Mean (SD) and proportions were used to summarize the characteristics of the study groups with and without RFPP-S. These were compared by analysis of variance. Categorical variables were compared using Cochran-Mantel-Haenszel statistics. Mixed regression analyses were used to assess changes in PTSD and maladaptive grief symptoms, RTLR, and psychiatric measures in RFPP-S compared with TAU. CIs and bias were calculated using a bootstrapping technique, where the bias was less than 2.0%. All statistical analyses were performed using SAS version 9.2 (SAS Institute Inc., Cary, North Carolina) and IBM SPSS version 27 (IBM Corp., Armonk, New York). The level of signiﬁcance was set at *p* < .05 (2-tailed). This study was approved and overseen by the Olive View–UCLA Education and Research Institute Institutional Review Board.

## Results

There was a 31.8% increase in the prevalence of youth treated for acute mental health crises at the PER during the COVID-19 pandemic (n = 1,567, September 2020 to November 2021) compared with a similar time frame before the pandemic (n = 1,189) (*p* < .05). Mean (SD) age of youth was 14.4 (2.4) years (range 7-18 years), and 70.13% (n = 1099) were female. The prevalence of youth with PTSD and suicidality visiting the PER was 48.7% higher during COVID-19 (n = 956, 61%) compared with the similar prepandemic time frame (n = 487,41%) (*p* < .05). During the COVID-19 pandemic, 40% of all youth with PTSD and suicidality treated at the PER experienced COVID-19–related PTSD, with 90% also experiencing grief reactions.

Mean (SD) age of youth was 14.5 (2.1) years, 30% were male, 75.8% had prior PER visits, and 21% had a prior psychiatry hospitalization. All youth were exposed to COVID-19–related losses (6.4% with parent loss, 40.8% with grandparent loss, 9.7% with friend/classmate loss, 32.2% with adult friend/family loss, and 10.5% with loss of teacher). Demographic and conventional risk factors are presented in Table 1. Increased levels of PTSD and grief symptoms, RTLR, guilt and loneliness, avoidance, and negative affectivity at baseline visits were observed in youth with COVID-19–related PTSD and grief (Table 1). There was a strong direct association between higher levels of RTLR with guilt feeling and suicidality.

**Table 1:** Clinical Characteristics of Study Subjects at Baseline and Follow-Up (FU)

Variables	RFPP-S group (n = 62)		Matched control group (n = 62)		*p*
Baseline					
	**Mean**	**(SD)**	**Mean (SD)**	**(SD)**	
Age, y	14.5	(2.1)	14.4	(2.2)	.9
	**n**	**(%)**	**n**	**(%)**	
Gender, female	43	(69.4)	44	(70.9)	.9
Prior PER visits	47	(75.8)	47	(75.8)	—
	**Mean**	**(SD)**	**Mean**	**(SD)**	
	2	(1)	2	(1)	—
	**n**	**(%)**	**n**	**(%)**	
Prior psychiatry hospitalization	13	(21)	13	(21)	—
	**Mean**	**(SD)**	**Mean**	**(SD)**	
	1.5	(0.5)	1.5	(0.5)	—
	**n**	**(%)**	**n**	**(%)**	
SSRI treatment	32	(51.6)	33	(53.2)	.9
Loss due to COVID-19					
Parent	4	(4)	4	(4)	—
Grandparent	25	(40.3)	26	(41.9)	.9
Friend/classmate	6	(9.7)	6	(9.7)	—
Adult friend/family	20	(32.2)	20	(32.2)	—
Teacher	7	(11.3)	6	(9.7)	.9
	**Mean**	**(SD)**	**Mean**	**(SD)**	
C-SSRS					
Suicide ideation	4.1	(0.7)	4.1	(0.7)	—
Suicide behavior	0		0		—
PHQ-9	22	(1)	21	(2)	.9
RTLR	19	(3)	19	(3)	—
Guilt and lonely feelings	9	(1)	9	(1)	—
Avoidance and negative affectivity	12	(1)	12	(1)	—
PTSD-RI score	44	(4)	44	(4)	—
PCBD Checklist score	76	(4)	76	(4)	—
Positive psychiatry battery					
PERMA scale score	19	(2)	18	(3)	.9
Gratitude score	8	(1)	9	(2)	.9
CD Resilience score	19	(1)	19	(2)	.8
PTGI score	10	(1)	10	(2)	.9
PACHIQ-R–Child	6.0	(0.7)	6.0	(0.9)	.9
PACHIQ-R–Parent	6.4	(0.7)	6.5	(0.9)	.9
Clinical outcome					
PER, days	1.9	(0.8)	4.7	(1.5)	.001
Day 2					
C-SSRS					
Suicide ideation	1.2	(0.7)	3.3	(1.1)	.001
Suicide behavior	0		0		—
PHQ-9	15	(1)	19	(2)	.001
RTLR	10	(2)	18	(2)	.001
Guilt and lonely feelings	5	(1)	9	(1)	.001
Avoidance and negative affectivity	8	(1)	11	(1)	.001
PTSD-RI score	32	(2)	40	(2)	.001
PCBD Checklist score	39	(3)	71	(4)	.001
	**n**	**(%)**	**n**	**(%)**	
SSRI treatment	35	(56.5)	34	(54.8)	.8
	**Mean**	**(SD)**	**Mean**	**(SD)**	
Positive psychiatry battery					
PERMA scale score	60	(1)	24	(3)	.001
Gratitude score	23	(2)	12	(2)	.001
CD Resilience score	62	(4)	14	(2)	.001
PTGI score	44	(3)	12	(2)	.001
PACHIQ-R–Child	7.9	(0.7)	6.5	(0.9)	.001
PACHIQ-R–Parent	7.9	(0.7)	6.4	(0.9)	.001
1-month FU					
	**n**	**(%)**	**n**	**(%)**	
1-wk postdischarge mental health FU	62	(100)	20	(32.2)	.001
1-mo rehospitalization	0		12	(19.3)	.001
	**Mean**	**(SD)**	**Mean**	**(SD)**	
1-mo FU C-SSRS	0.7	(0.6)	1.9	(1.1)	.001

Note: CD Resilience = Connor-Davidson Resilience Scale; C-SSRS = Columbia–Suicide Severity Rating Scale; PACHIQ-R = Parent-Child Interaction Questionnaire–Revised; PCBD = Persistent Complex Bereavement Disorder; PER = psychiatric emergency room; PERMA = Positive Emotion, Engagement, Relationships, Meaning, and Accomplishment; PHQ-9 = Patient Health Questionnaire; PTGI = Posttraumatic Growth Inventory; PTSD-RI = UCLA Child/Adolescent PTSD Reaction Index for *DSM-5;* RFPP-S = reminder-focused positive psychiatry and suicide prevention; RTLR = reactivity to trauma and loss reminders; SSRI = selective serotonin reuptake inhibitor.

On day 2, there was a significant reduction in COVID-19–related maladaptive grief symptoms and PTSD, RTLR, guilt feelings, negative affectivity, and avoidance in the RFPP-S group, but not in TAU, compared with baseline (*p* = .001). There was a significant improvement in the parent–child relationship in both groups, significantly more pronounced with RFPP-S (*p* = .001). C-SSRS scores were reduced substantially in both groups compared with baseline (*p* < .05), which was more robust in the RFPP-S group (*p* = .0001). A significant reduction in the length of stay in the PER was noted in the RFPP-S group compared with TAU (*p* = .001) (Table 1).

Table 1 shows a robust difference in postdischarge mental health follow-up in the RFPP-S group (100%) compared with TAU (51.6%) (*p* < .05). Further, RFPP-S, but not TAU, was associated with sustained reduced C-SSRS with no PER visit or psychiatry hospitalization within 1 month postdischarge.

**Figure 2:**
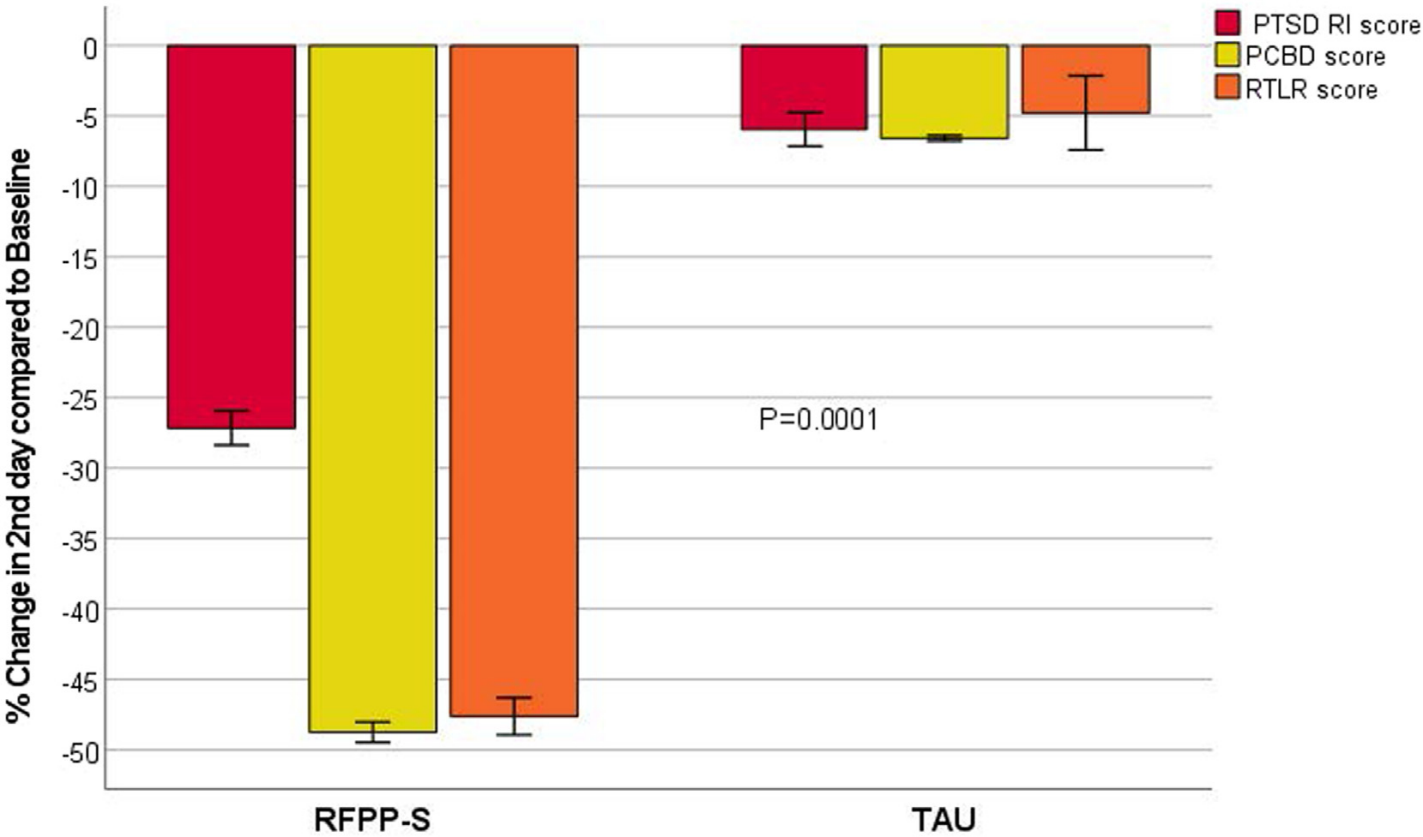
Impact of a Grief-Enhanced Trauma-Informed Intervention in Youth With Maladaptive Grief and Posttraumatic Stress Disorder (PTSD) With Suicidality ***Note:****A significant decrease was noted in reactivity to trauma stress/loss reminders (RTLR), core PTSD symptoms measured by the UCLA Child/Adolescent PTSD Reaction Index for* DSM-5 *(PTSD-RI), and bereavement symptoms measured by the Persistent Complex Bereavement Disorder (PCBD) Checklist at day 2 in response to the reminder-focused positive psychiatry and suicide prevention (RFPP-S) grief-enhanced trauma-informed intervention compared with treatment as usual (TAU) (*p *= .0001).*

Figure 2 shows a significant difference in the reduction of COVID-19 maladaptive grief (42%) and PTSD (20%) symptoms and a decrease in RTLR (42%) in the RFPP-S group at day 2 compared with TAU (*p* = .0001).

**Figure 3:**
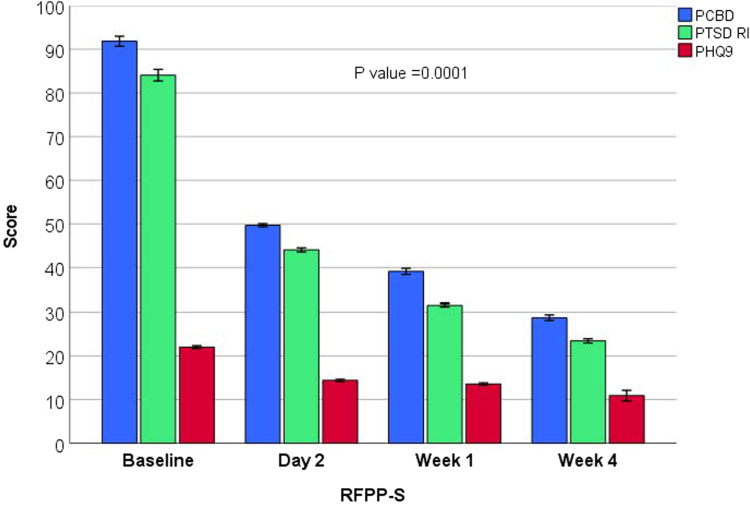
Postdischarge Effect of Grief-Enhanced Trauma-Informed Emergency Department Intervention on Core Symptoms of Youth With Maladaptive Grief and Posttraumatic Stress Disorder (PTSD) With Suicidality ***Note:****There was a sustained decrease in core PTSD symptoms measured by the UCLA Child/Adolescent PTSD Reaction Index for* DSM-5 *(PTSD-RI), bereavement symptoms measured by the Persistent Complex Bereavement Disorder (PCBD) Checklist, and depressive symptoms measured by the Patient Health Questionnaire (PHQ-9) in response to the reminder-focused positive psychiatry and suicide prevention (RFPP-S) grief-enhanced trauma-informed intervention at 4-week follow-up (*p *= .0001).*

Table 2 demonstrates a significant, sustained reduction in C-SSRS scores from high risk for suicidal ideation to low risk (*p* < .05). In addition, Figure 3 reveals a significant improvement in PTSD-RI and PCBD Checklist scores in response to RFPP-S from day 2 to week 4. There was a significant direct association between reduced guilt and loneliness, avoidance, negative affectivity, and RTLR, with decreased suicidality, grief, and PTSD symptoms (*r*^2^ = 0.83, *p* = .001).

**Table 2:** Changes in Core Grief and Posttraumatic Stress Disorder Symptoms, Well-Being Indices, and Parent–Child Interactions in Reminder-Focused Positive Psychiatry and Suicide Prevention (RFPP-S) at Day 2 Compared With Matched Control

Model	Matched control	RFPP-S	*p*
Change in severity of RTLR^a^	1.0 (reference)	0.32 (0.22-0.42)	.001
Change in PTSD-RI score^a^	1.0 (reference)	0.62 (0.24-0.76)	.005
Change in PCBD Checklist score^a^	1.0 (reference)	0.28 (0.15-0.42)	.001
Change in C-SSRS score	1.0 (reference)	0.48 (0.29-0.63)	.001
Change in PERMA scale score^a^	1.0 (reference)	3.25 (2.95-4.12)	.001
Change in CD Resilience score^a^	1.0 (reference)	3.96 (3.32-4.31)	.001
Change in PTGI score^a^	1.0 (reference)	3.62 (3.04-4.21)	.001
Change in PACHIQ-R score^a^	1.0 (reference)	2.29 (1.59-5.12)	.005

Note: CD Resilience = Connor-Davidson Resilience Scale; C-SSRS = Columbia–Suicide Severity Rating Scale; PACHIQ-R = Parent-Child Interaction Questionnaire–Revised; PCBD = Persistent Complex Bereavement Disorder; PERMA = Positive Emotion, Engagement, Relationships, Meaning, and Accomplishment; PHQ-9 = Patient Health Questionnaire; PTGI = Posttraumatic Growth Inventory; PTSD-RI = UCLA Child/Adolescent PTSD Reaction Index for *DSM-5;* RTLR = reactivity to trauma and loss reminders.

a Standard deviation change from baseline to day 2 mixed regression.

**Figure 4:**
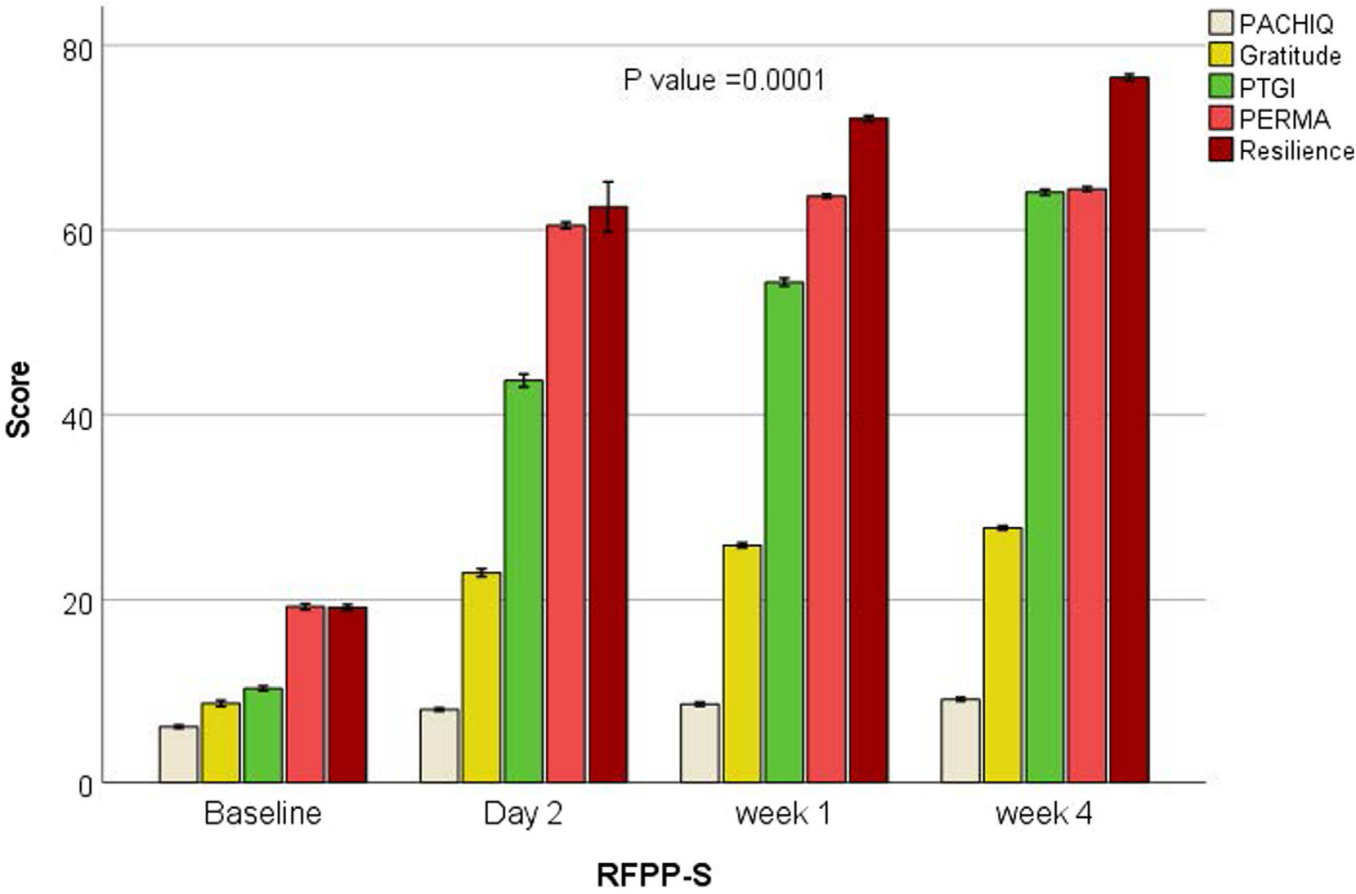
Postdischarge Effect of Grief-Enhanced Trauma-Informed Emergency Department Intervention on Mental Well-Being, Gratitude, Posttraumatic Growth, and Resilience in Youth With Maladaptive Grief and Posttraumatic Stress Disorder (PTSD) With Suicidality ***Note:****There was a sustained improvement in mental well-being, resilience, posttraumatic growth, gratitude, and parent–child interactions in response to the reminder-focused positive psychiatry and suicide prevention (RFPP-S) grief-enhanced trauma-informed intervention at 4-week follow-up (*p *= .0001). PACHIQ-R = Parent-Child Interaction Questionnaire–Revised; PERMA = Positive Emotion, Engagement, Relationships, Meaning, and Accomplishment; PTGI =* Posttraumatic Growth Inventory.

Figure 4 reveals a significant increase in well-being (273%), engagement and connectedness with caregivers (250%), resilience and adaptability (325%), posttraumatic growth, and enhanced coping skills (415%) in response to RFPP-S compared with baseline (*p* < .05) that was maintained at 1 month postdischarge. We found a significant direct association between the reduction of COVID-19–related RTLR with increased engagement and well-being, resilience and flexibility, PTGI and adaptability, and parent–child interactions (*r*^2^ = 0.94, *p* = .001).

Table 3 shows that the relative risk of each standard deviation decreased with the severity of RTLR, PCBD Checklist, PTSD RI, and C-SSRS scores on day 2 (0.32, 0.28, 0.62, and 0.48) in youth with COVID-19–related maladaptive grief reactions and PTSD symptoms receiving RFPP-S compared to TAU (*p* < .05), with sustained improvement at week 4. In addition, the relative risk of increase in engagement and well-being, resilience, PTGI and adaptability, and parent–child interactions on day 2 was 3.25, 3.96, 3.62, and 2.29 with RFPP-S compared with TAU (*p* < .05), with sustained enhancement at week 4.

**Table 3:** Clinical Characteristics of Study Subjects at Baseline and Follow-Up (FU) and Changes in Response to Reminder-Focused Positive Psychiatry and Suicide Prevention (RFPP-S)

Variable	Baseline (n = 62)		Day 2 PER (n = 62)		Week 1 (n = 62)		1-mo FU (n = 62)		*p*
	n	(%)	n	(%)	n	(%)	n	(%)	
SSRI treatment	32	(51.6)	35	(56.5)	35	(56.5)	35	(56.5)	—
	**Mean**	**(SD)**	**Mean**	**(SD)**	**Mean**	**(SD)**	**Mean**	**(SD)**	
PHQ-9	22	(1)	15	(1)	13	(1)	11	(2)	.001
C-SSRS									
Recent suicide ideation	4.1	(0.7)	1.2	(0.7)	1	(0.7)	0.7	(0.6)	.001
Recent suicidal behavior	0		0		0		0		—
RTLR	19	(3)	10	(2)	9	(1)	6	(1)	.001
Guilt and lonely feelings	9	(1)	5	(1)	4	(1)	3	(1)	.001
Avoidance and negative affectivity	12	(1)	8	(1)	6	(1)	4	(1)	.001
PTSD-RI score	44	(4)	32	(2)	23	(2)	16	(2)	.001
PCBD Checklist score	76	(4)	39	(3)	30	(3)	25	(2)	.001
PP batteries							1		
PERMA scale score	19	(2)	60	(1)	63	(1)	64	(1)	.001
Gratitude score	8	(1)	23	(2)	26	(1)	28	(1)	.001
CD Resilience score	19	(1)	62	(4)	72	(2)	76	(2)	.001
PTGI score	10	(1)	44	(3)	54	(2)	64	(2)	.001
PACHIQ-R–Child	6.0	(0.7)	7.9	(0.7)	8.5	(0.8)	8.9	(0.8)	.001
PACHIQ-R–Parent	6.4	(0.7)	7.9	(0.7)	8.5	(0.8)	8.9	(0.7)	.001
	**%**		**%**		**%**		**%**		
Postdischarge MH F/U	—		—		100		100		—
PER visit									
Prior visits	—		100		100		100		—
	**n**		**n**		**n**		**n**		
Visits	—		0		0		0		—
Psychiatric hospitalization	—		0		0		0		—
	**%**		**%**		**%**		**%**		
Prior hospitalization	—		0		0		0		—
	**n**		**n**		**n**		**n**		
Hospitalizations	—		0		0		0		—

Note: PTSD-RI: score range 0-124, higher scores (≥36) indicating PTSD symptoms; RTLR: score range 0-36); PCBD Checklist: score range 0-156, higher scores (≥30) indicating grief symptoms); PHQ-9: score range 0-27, higher scores (≥20) indicating severe depressive symptoms); C-SSRS: score range 0-5, higher scores (≥3) indicating moderate to severe suicidality; PERMA: score range 1-10, higher scores (≥5) indicating higher levels of mental well-being); CD Resilience: score range 0-100, higher scores (≥50) exhibiting higher levels of resilience and adapting new skillsets to bounce back); PTGI: score range 0-1, higher scores (≥4) indicating higher levels of growth with enhancement of coping skills; PACHIQ-R: score range 0-10, higher scores (≥7) indicating higher levels of parent–child interactions and socialization; Gratitude survey: score range 0-42, higher scores (≥24) indicating higher levels of engagement and self-appreciation). CD Resilience = Connor-Davidson Resilience Scale; C-SSRS = Columbia–Suicide Severity Rating Scale; PACHIQ-R = Parent-Child Interaction Questionnaire–Revised; PCBD = Persistent Complex Bereavement Disorder; PER = psychiatric emergency room; PERMA = Positive Emotion, Engagement, Relationships, Meaning, and Accomplishment; PHQ-9 = Patient Health Questionnaire; PP = positive psychiatry; PTGI = Posttraumatic Growth Inventory; PTSD = posttraumatic stress disorder; PTSD-RI: UCLA Child/Adolescent PTSD Reaction Index for DSM-5; RTLR = reactivity to trauma and loss reminders; SSRI = selective serotonin reuptake inhibitor.

## Discussion

The present study supports the following novel findings:

1. During the pandemic, the rate of adolescents with COVID-19–related maladaptive grief reactions and PTSD symptoms with suicidality admitted to the PER significantly increased.
2. A brief grief-enhanced trauma-informed safety preventive emergency department intervention was associated with ameliorating maladaptive grief and PTSD reactions and RTLR and rapid stabilization of acute crisis with enhanced safety.
3. A direct link was found between a reduction in loss-related guilt and loneliness, negative affectivity and avoidance, and RTLR.
4. General mental well-being, positive engagement, posttraumatic growth, and parent–child interactions improved in response to intervention.
5. All patients completed the discharge mental health visit; no patients had suicidality, a subsequent psychiatry emergency visit, or psychiatric hospitalization within 30 days postdischarge.

The COVID-19 pandemic has been associated with multiple losses, including the death of loved ones; significant life changes in COVID-19 survivors; loss of social/physical freedom, relationships, and social support; parents losing employment, home, financial security, and health care; and loss of independence and sense of the future.^22^, ^23^, ^24^, ^25^, ^26^, ^27^ As a result of prolonged and disabling grief, losses, and traumatic experiences, more individuals are placed at risk of PTSD and maladaptive grief reactions with impairment in physical and mental well-being.^23^^,^^24^ A recent cross-sectional study revealed that the total mortality rate of COVID-19 was 6.8% in people with a mental disorder compared with 5.3% in people without a mental disorder.^3^ Multiple studies have reported that youth visits to PERs, especially youth with trauma exposure and self-harm behavior with suicidality, are increasing across the United States.^6^^,^^10^^,^^28^, ^29^, ^30^, ^31^, ^32^

Many individuals with bereavement struggle with a troubling sense of death; their own role, responsibility, and guilt for not being able to prevent the death; lack of adequate social support; and increased risk of suicidality.^33^ Prior studies have revealed a strong association between reactivity to loss reminders and suicidality among adolescents in the context of their desire to reunite with their deceased loved ones.^34^, ^35^, ^36^ The rate of bereavement among youth due to parental loss increased to 20.2% during the COVID-19 pandemic compared with 17.5% before the pandemic.^37^ The shocking nature of death due to COVID-19 has been shown to be associated with increased feelings of guilt, disturbances in interactions with loved ones, and lack of social gathering and support, making adaptation more difficult for survivors.^37^, ^38^, ^39^ Bereaved individuals with COVID-19–related losses have demonstrated more severe grief symptoms and lower social support compared with other types of natural losses.^38^^,^^39^

For more than half of the youth seeking care in the PER, this was the first point of contact with mental health care services. More than half of the youth who visited the PER had issues related to PTSD.^11^^,^^12^^,^^32^^,^^40^, ^41^, ^42^, ^43^, ^44^ This has become more pronounced during the COVID-19 pandemic. The current study shows that the prevalence of youth who visited the PER with PTSD and suicidality during the COVID-19 pandemic increased by 61%, including 40% with COVID-19–related PTSD and grief symptoms.

Recent studies have demonstrated the efficacy of brief suicide risk screening and intervention, including SAFETY-A, to prevent suicide with higher adherence to postdischarge follow-up appointments, reducing suicide risk and admission rate.^11^^,^^12^^,^^40^, ^41^, ^42^, ^43^, ^44^, ^45^, ^46^ Prior studies revealed the positive outcome of self-compassion, self-forgiveness, resilience, and gratitude in reducing bereavement-related emotional distress and suicide behavior. RFPP-S intervention for both caregivers and adolescents at the PER has resulted in acute crisis stabilization, enhanced child–parent interactions, positive engagement, enhanced emotional regulation, improved school attendance and performance, and no recurrence of symptoms or psychiatric hospitalization within 6 months.^47^

The current study suggests that a brief grief-enhanced trauma-informed intervention delivered in a PER can provide benefit for youth with bereavement and PTSD in helping to reveal and discuss their private loss experience and grief reactions, to focus on enhancing contextual discrimination, emotional regulation, and adaptive coping strategies by shifting attention from intrusive memories during exposure to trauma/loss reminders to the meaning of reactions, feelings, thoughts, goals, and choices through self-compassion and gratitude. Further, this brief PER intervention addresses avoidance, negative affect, negative cognitions, separation, and identity distress using positive perceptual-sensory input, promoting a mindset with connectedness and empathy through compassionate ABCD engagement, developing future aspirations and meaning in the aftermath of loss, and promoting safety. A significant improvement of PTSD and grief symptoms in response to RFPP-S was noted, which was more robust with the improvement of loss reminder and grief symptoms. The current study confirms prior studies and, for the first time, demonstrates that a brief grief-enhanced trauma-informed PER intervention can provide youth opportunities to communicate their experience of loss and related reactions, reengage with their families through enhanced positive caregiver support, and develop a positive plan for the future with a sense of enhanced personal identity; develop coping skills to manage RTLRs, using positive self-talk, emotional regulation, and distress tolerance skills to address negative affectivity and avoidance; and develop safety plans. RFPP-S was associated with improving RTLR, PTSD, and grief symptoms and enhancing acute crisis stabilization of youth with COVID-19–related PTSD and grief with suicidality. In addition, improving COVID-19–related RTLR is linked to improved mental well-being, resilience, positive engagement, posttraumatic growth, parent–child interactions, and favorable clinical outcomes in response to this brief PER intervention.

This study has several limitations. It was a single-center, nonrandomized retrospective observational analysis of a cohort of adolescents with comorbid COVID-19–related grief and PTSD with suicidality. RFPP-S treatment was done by one provider. Thus, data acquisition for this study was unblended. Although there was a comparison group, there was no placebo treatment control. A significant number of adolescents with COVID-19–related grief and PTSD with suicidality had received an adequate selective serotonin reuptake inhibitor before PER admission and were kept on home doses without change in the first 48 hours of the PER visit. The change in selective serotonin reuptake inhibitor and/or dosage after day 2 at the Olive View UCLA PER was not available for this study. Further prospective studies and randomized controlled clinical trials are warranted to evaluate the effects of RFPP-S on early detection and optimal management of adolescents with COVID-19–related grief and PTSD with suicidality.

In conclusion, COVID-19–related maladaptive grief and PTSD reactions with suicidality were found to be prevalent among adolescents who visited the PER. The study demonstrates the feasibility and effectiveness of delivering a grief-enhanced trauma-informed safety preventive intervention in a PER setting. This intervention was associated with a robust reduction in maladaptive grief and PTSD reactions, reduced RTLR, reduced suicidality, rapid stabilization of acute crisis, improved distress tolerance, adherence to post-PER follow-up visits and treatments, and favorable clinical outcomes. In addressing the continued concern for suicidality among youth, special attention must be given to grief related to COVID-19–related losses. Parental and PER therapeutic support holds promise for an important public health strategy for COVID-19–related grieving suicidal youth.

The promising results underscore the importance of addressing both loss exposure and maladaptive grief, while adopting a trauma-informed approach to the evaluation and management of adolescent suicide risk.^48^ The findings add to the growing literature suggesting that a PER is an important intervention site.^48^ The positive outcomes will need replication in larger-scale controlled studies, but the direction and strength of the findings are not surprising, given the potential benefit of bringing therapeutic attention to unspoken grief, suicidal reunion thoughts, adolescent guilt, and previously unsupported confrontations with trauma and loss reminders. The reported improvements were obtained in a PER that serves a large, diverse population hard hit by COVID-19, suggesting one path toward addressing the vast disproportionality of COVID-19 caregiver and family deaths for children and adolescents in the United States.^49^

## CRediT authorship contribution statement

**Naser Ahmadi:** Writing – review & editing, Writing – original draft, Visualization, Validation, Supervision, Software, Resources, Project administration, Methodology, Investigation, Funding acquisition, Formal analysis, Data curation, Conceptualization. **Julie B. Kaplow:** Writing – review & editing, Visualization, Validation, Resources, Conceptualization. **Alan Steinberg:** Writing – original draft, Visualization, Validation, Supervision, Resources, Investigation, Formal analysis. **James T. McCracken:** Writing – review & editing, Visualization, Supervision, Resources, Methodology. **Steven J. Berkowitz:** Writing – review & editing, Validation, Methodology, Data curation, Conceptualization. **Robert S. Pynoos:** Writing – original draft, Visualization, Validation, Supervision, Resources, Methodology, Formal analysis, Data curation, Conceptualization.
